# Effects of Brazilian scorpion venoms on the central nervous system

**DOI:** 10.1186/s40409-018-0139-x

**Published:** 2018-01-23

**Authors:** Ana Leonor Abrahão Nencioni, Emidio Beraldo Neto, Lucas Alves de Freitas, Valquiria Abrão Coronado Dorce

**Affiliations:** 1 Laboratory of Pharmacology, Butantan Institute, Av. Dr. Vital Brasil, 1500, São Paulo, SP 05503-900 Brazil; 2 Graduation Program in Sciences – Toxinology, Butantan Institute, São Paulo, SP Brazil

**Keywords:** Brazilian scorpions, Central nervous system, Scorpion venom, Scorpion toxins

## Abstract

In Brazil, the scorpion species responsible for most severe incidents belong to the *Tityus* genus and, among this group, *T. serrulatus*, *T. bahiensis*, *T. stigmurus* and *T. obscurus* are the most dangerous ones. Other species such as *T. metuendus*, *T. silvestres, T. brazilae*, *T. confluens*, *T. costatus*, *T. fasciolatus and T. neglectus* are also found in the country, but the incidence and severity of accidents caused by them are lower. The main effects caused by scorpion venoms – such as myocardial damage, cardiac arrhythmias, pulmonary edema and shock – are mainly due to the release of mediators from the autonomic nervous system. On the other hand, some evidence show the participation of the central nervous system and inflammatory response in the process. The participation of the central nervous system in envenoming has always been questioned. Some authors claim that the central effects would be a consequence of peripheral stimulation and would be the result, not the cause, of the envenoming process. Because, they say, at least in adult individuals, the venom would be unable to cross the blood-brain barrier. In contrast, there is some evidence showing the direct participation of the central nervous system in the envenoming process. This review summarizes the major findings on the effects of Brazilian scorpion venoms on the central nervous system, both clinically and experimentally. Most of the studies have been performed with *T. serrulatus* and *T. bahiensis*. Little information is available regarding the other Brazilian *Tityus* species.

## Background

Approximately 1500 scorpion species, distributed among 18 families, are described worldwide [1]. From these species, only nearly 30, belonging to the Buthidae family, are dangerous for humans and are responsible for serious envenoming or death [2–5].

In Brazil, from about 160 scorpion species that occur in the country, those belonging to *Tityus* genus are responsible for severe incidents. *T. serrulatus, T. bahiensis, T. stigmurus and T. obscurus* are the most dangerous ones found in the country. Other species, such as *T. metuendus, T. silvestres, T. brazilae, T. confluens, T. costatus, T. fasciolatus, T. neglectus, T. aba, T. anneae, T. carvalhoi, T. cylindricus, T. kuryi, T. maranhensis, T. martinpaechi, T. mattogrossensis, T. melici, T. pusillus, and T. trivittatus*, also occur, but the incidence and severity of accidents caused by them are lower [6–10].

*Tityus serrulatus* is the Brazilian scorpion that is responsible for the most severe accidents, with mortality rates of approximately 1% among children and elderly people [11]. This species is widely distributed throughout the country, reaching the states of São Paulo, Minas Gerais, Bahia, Espírito Santo, Goiás, Paraná and Rio de Janeiro [4, 12]. Similarly, *T. bahiensis* is widely distributed throughout the country, except for northern regions, and it is responsible for most of the accidents in the Southeast region [2, 4]. *T. stigmurus* is distributed predominantly in the Northeastern region of the country, where it is the main cause of accidents [4]. In the Brazilian Amazon, the main species with medical interest are *T. obscurus*, *T. metuendus* and *T. silvestris* [11, 13].

The main effects caused by scorpion venoms – such as myocardial damage, cardiac arrhythmias, pulmonary edema and shock – are mainly due to the release of mediators from the autonomic nervous system [13]. On the other hand, some evidence show the participation of the central nervous system and of the inflammatory system in the process [14–30].

The participation of the central nervous system in the envenoming process has always been questioned. According to Freire-Maia and Campos [31], the central effects would be the result and not the cause of the envenoming process, since the venom would be unable to cross the blood-brain barrier. On the other hand, Ismail et al. [32, 33] believe in the direct participation of the central nervous system in the process, especially in very young individuals, where the blood brain barrier would not be fully formed.

Worldwide, some clinical reports have indicated the involvement of the central nervous system in the effects of the venom. Nagaraja et al. [34], in a study carried out in India, reported two cases of stroke after a scorpion sting. Barthwal et al. [35] also reported a case of brain infarct after myocarditis and pulmonary edema, after a scorpion sting. Fernandez-Bouzas et al. [36] reported two children with severe neurological complications after scorpion stings. Tracker et al. [37] reported a case of multiple cerebral infarcts, limb ischemia and bilateral optic neuropathy due to scorpion (possibly a *Buthus tumulus*) envenoming. Gadwalkar et al. [38] demonstrated a rare case of extensive cerebellar infarction following a scorpion sting caused by the vasculotoxic action of the scorpion venom. Prasad et al. [39] reported a case of ischemic infarction of the cerebral cortex in a child suffering from scorpion envenoming. Sigirci et al. [40] demonstrated cerebellar and cerebral infarctions with corpus callosum involvement and bilateral cerebral atrophy with subdural hemorrhage in an 8-month-old girl stung by a *Leiurus quinquestriatus.* Unfortunately, most of the scorpions that caused the accidents were not identified.

In Brazil, cerebrovascular complications after scorpion stings are rare. Few cases have been described in the literature. Bonilha et al. [41] reported a case of a child who developed epilepsy due to a destructive brain lesion after a sting by *T. serrulatus*. Oliveira et al. [42] reported neurological alterations such as hemiplegia, paralysis of the facial nerve and cerebral edema in a 10-year-old girl who was stung by an unidentified scorpion. Seizures and hemorrhagic stroke on the frontal lobe were described in a woman stung by *T. serrulatus* [43]. Marrone et al. [44] described the first case of posterior reversible encephalopathy syndrome in a 13-year-old boy stung by *T. bahiensis*. Bucaretchi et al. [45] reported a fatal envenoming involving multiple, extensive brain infarcts in a patient with a previous diagnosis of essential thrombocythemia who was stung by *T. serrulatus*.

Moreover, experimental studies performed mainly with *T. serrulatus* and *T. bahiensis* have demonstrated the central effects of the scorpion venoms and toxins [17–19, 46].

This review aims to provide an update of clinical and experimental findings on the effects of Brazilian scorpion venoms on the central nervous system.

### *Tityus serrulatus*

**Fig. 1:**
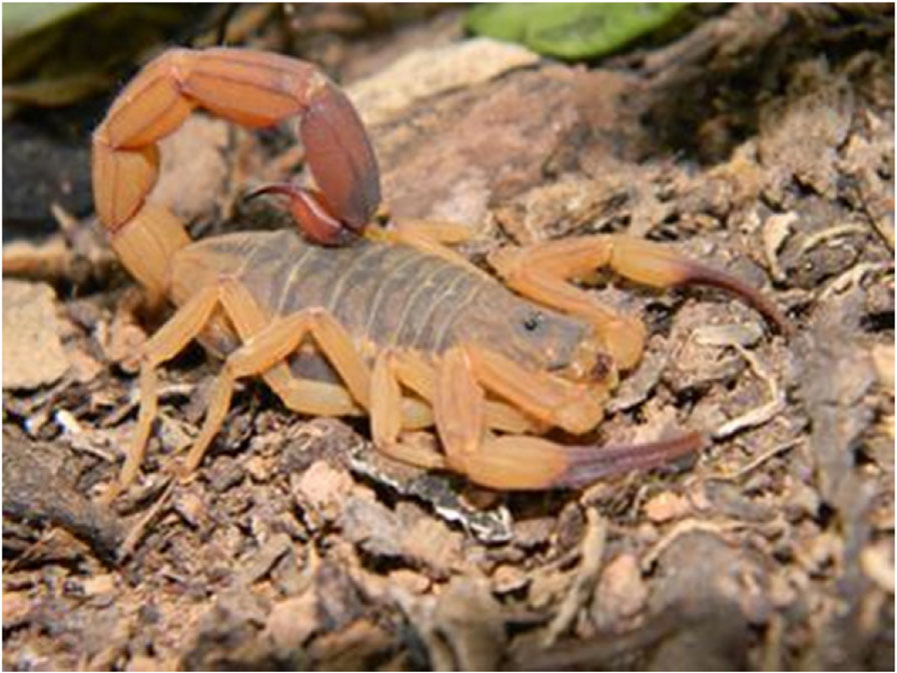
*Tityus serrulatus.* Known as the yellow scorpion, *T. serrulatus* is 5 to 7 cm long, with the third and fourth segments of the metasoma serrated, and parthenogenetic reproduction. Source: Brazilian Ministry of Health [6]. Image copyright by Denise Cândido, reproduced with permission

*T. serrulatus* is the most known Brazilian scorpion (Fig. 1) and its venom has been extensively studied. Lutz and Mello described this species for the first time in Brazil in 1922. Its reproduction is parthenogenetic [5].

Clinically, it is responsible for the majority of the accidents in the country [12, 20]. Local pain is the primary local manifestation, and this type of accident is classified as mild [47, 48]. In moderate cases, cardiac effects, vomiting, abdominal pain, agitation, hypersalivation, fever, priapism, and hyperglycemia occur, whereas in severe cases, cardiovascular, pulmonary, gastrointestinal and metabolic complications appear, in addition to neurological symptoms [47, 48]. Central effects such as coma and convulsion rarely appear, therefore there are only few cases described in the literature [41, 43, 45].

Experimentally, studies on *T. serrulatus* venom started in the 1960s by Gomez and Diniz, when they reported the first fractionation process and the first fraction obtained was named “tityustoxin” [49]. For a long time, this component was considered a purified toxin. However, the improvement of the purification methodology showed it is a “pool” constituted of several peptides [50]. Since then, this pool has been designed as “tityustoxin” (in quotation marks) in order to differentiate it from the purified tityustoxin (without quotation marks) described later [51–53]. One of the first studies performed with “tityustoxin” showed cardiovascular and respiratory effects after intravenous injection [54]. The intracerebral injection induced similar cardiorespiratory alterations, in addition to neurological alterations, such as tremors, hyper-reactivity, extensor rigidity and convulsions [54].

Since the first purification processes, several toxins have been isolated and sequenced and some of their biological effects have been characterized [15, 52, 53, 55–64].

Throughout more than 50 years, many studies have tried to explain the action of this venom and its components on the central nervous system. Although Revelo and collaborators [65] have not detected *T. serrulatus* venom in the central nervous system after subcutaneous injection in an immunoenzymatic assay, a number of studies have demonstrated the ability of this venom, or part of it, to cross the blood-brain barrier and to reach the central nervous system [66–69].

Studies performed with the whole venom have demonstrated its inhibitory effect on the sodium-dependent amino acid uptake in synaptosomes and synaptic membrane vesicles, thus affecting the absorption capacity of these amino acids [70]. Pre-treatment with the venom alters the threshold and intensity of seizures in different animal models of epilepsy. The intrahippocampal injection in rats is able to promote behavioral changes and epileptiform activity [71]. Intravenous or intracerebral injection alters the level of neurotransmitters in different regions of the brain, revealing a connection between the action of this venom and GABA and dopamine [14]. Intraperitoneal injection induces electrographic and behavioral alterations in rats [17]. In studies with isolated preparations of rat brain synaptosomes, it has been shown that this venom is not able to alter the glutamate uptake; however, it promotes an inhibition of the GABA and dopamine uptake caused by the action of the venom on the Na^+^ channels [72].

Its actions on the central nervous system have generated great interest in the identification of isolated components to better elucidate the action of *T. serrulatus* venom on the central nervous system. Several toxins have already been isolated and their central effects have been described.

Some of the major toxins that affect the central nervous system are:

- Ts1, also known as TsTX-I, Ts VII or toxin γ, is the most abundant and the most toxic component isolated from *T. serrulatus* venom, corresponding to about 16% of the soluble fraction of the venom [73–75]. It acts as a classical β-toxin [75], modulating the activation process of sodium channels Nav1.6 and Nav1.3 in the negative potential direction, causing the opening of the channels at the resting potential. However, electrophysiological studies of Ts1 in Nav channels of insects resemble the effect of a typical site 3 toxin following a bell-shaped voltage dependence that does not occur with other β toxins. Additionally, Ts1 inhibits the sodium current through Nav 1.5 channels without altering the activation or steady-state inactivation curves [76]. The intracerebral injection of Ts1 in rats causes epileptiform discharges and wet dog shake behavior, and it is also able to cause paralysis of the hind limbs and severe respiratory distress followed by death [46], without altering the intrahippocampal concentration of glutamate [77]. The injection of Ts1 affects the neuroimmunological system, increasing the level of tumor necrosis factor α (TNF-α) and interferon gamma (IFN-γ) in the rat brain [77, 78].
- Ts3, also known as TsTX, toxin IV-5, TS-8F or tityustoxin, is considered the most lethal α-toxin, component of the *T. serrulatus* venom [79, 80]. Even small doses of this toxin may have a lethal effect in adult rats when injected directly into the brain [81, 82]. According to Guidine et al. [68], the toxin affects brainstem structures involved in neurovegetative control, such as cardiovascular and respiratory functions. When subcutaneously injected, the toxin crosses the blood-brain barrier and reaches these centers [69]. The basic action of this toxin is to delay the inactivation of voltage-dependent sodium channels, which increases the permeability of the cell membrane to sodium, thereby, increasing the release of the neurotransmitters [14, 83–85]. After intrahipocampal injection, Ts3 promotes the release of glutamate, causing epileptic-like discharges and neuronal loss in CA1, CA3 and CA4 hippocampal areas [15, 86, 87]. Four months after the injection, neuronal loss and mossy fiber sprouting were still observed in the supragranular layer of the dentate gyrus in rats [88]. TsTX also evokes glutamate release from cortical synaptosomes, and calcium is involved in this release [85, 89]. According to Silva et al. [90], the epileptiform discharges caused by TsTX are correlated with cardiac arrhythmias. Intracerebral injections of Ts3 can still produce severe lung edema, lead to a cerebral inflammatory process with higher levels of TNF-α and induce an increase in the microvascular leukocyte recruitment [78, 91].
- Ts4, also known as TsTX-VI, was described as a less toxic toxin, but it can cause allergic reaction (lachrymation, spasm in mouse hind paws) and it is capable of causing the release of neurotransmitters such as glutamic acid and GABA from rat brain synaptosomes [92]. This toxin specifically inhibits the rapid inactivation of the Nav1.6 channel [63].
- Ts5 is an α-neurotoxin capable of delaying the inactivation of voltage-dependent sodium channels [57, 58, 72]. It shows high toxicity and constitutes about 2% of the soluble fraction of the venom [74]. It acts specifically on channels Nav1.2, Nav1.3, Nav1.4, Nav1.5, Nav1.6 and Nav1.7, inhibiting rapid inactivation [62]. It is capable of causing the release of catecholamines and the reduction of GABA and dopamine in vitro, because of the depolarization, involving voltage-dependent sodium channels [72, 74]. Ts5 also acts as a proinflammatory toxin, inducing the production of TNF-α and IL-6 [62].

Other important toxins, whose effects on the central nervous system have not been directly demonstrated yet, should not be ruled out due to their action on ion channels, essential elements for the functioning of the central nervous system.

Among the toxins acting on sodium channels is Ts2, also known as TsTX-III or III-8, which has been classified as both an α- and β-toxin [53, 56]. Ts2 inhibits the rapid inactivation of some sodium channels, but does not affect others [93]. It represents the newest member of a small group of toxins with the structural features of β-toxins but displaying α-like activity [94]. Ts17 and Ts18 toxins have been described based on transcriptomic studies from venom glands, and neurotoxic activities were attributed to these toxins [95]. Ts17 was classified as a toxin that acts on sodium channels, since its sequence has about 86% of identity with the Ts5 toxin [95]. Ts18 is also classified as a toxin that acts on sodium channels, due to the high degree of identity (63%) with the U1-buthitoxina-Hj1a toxin, a sodium channel toxin isolated from the venom of the black scorpion *Hottentotta judaicus* [95, 96].

Among the toxins that act on potassium channels is Ts6, also known as TsTX-IV, which is able to block calcium activated potassium channels of high conductance. Ts6 showed a high blocking effect on Kv1.2, Kv1.3 and Shaker IR channels and was capable of blocking, with low efficiency, the channels Kv1.1, Kv1.5, Kv1.6, Kv4.3, Kv7.1, Kv7.2, Kv7.4 and hERG [97, 98]. It has a high capacity to interact with different subtypes of K^+^ channels with different affinities [99]. According to Arantes et al. [52], this toxin induces a release of noradrenaline.

Ts7, also known as TsTX-Kα or tityustoxin K-α, has been classified as a potent and selective potassium channel blocker toxin [100, 101], which partially inactivates K^+^ currents in dorsal root ganglion neurons of rats [102]. Ts7 showed a high and significant blocking effect on Kv1.1, Kv1.2, Kv1.3, Kv1.6 and Shaker [98]. Some years ago, studies have classified this toxin as a simple blocker of Kv1.3 channels [103].

Ts8, also known as tityustoxin K-β or TsTX-K β, is a 60-amino-acid-residue peptide and can be classified as β-KTX (toxins acting on potassium channels) [64], which means that it selectively blocks voltage-gated noninactivating K^+^ channels in synaptosomes [104]. Since it shows a very different sequence from the standard observed for toxins that act on K^+^ channels (toxins that have between 23 and 42 amino acid residues), it was classified as the first toxin from the β-KTx subfamily [94].

Ts8 presents a specific inhibiting effect on Kv4.2, showing a reversible inhibition [105]. Ts9, also known as TsKappa, has been described as an active toxin on calcium-activated small conductance potassium channels [59]. Cologna et al. [75] classified the peptides TsPep1, TsPep2 and TsPep3 described by Pimenta et al. [106] as Ts11, Ts12 and Ts13, respectively. These peptides are formed by four disulfide bridges, and their structural characteristics point out that they are active on K^+^ channels, on the basis of a functional analysis evidencing these toxins as preferential Kv blockers. Due to the poor percentage of identity with the other KTxs, Cremonez et al. [107] suggested that they can be regarded as the first members of a new subfamily of KTxs, called ε-KTx.

Ts15, also known as α-KTx21, is capable of blocking potassium channels in a nanomolar range [60, 108]. In 2013, Verano-Braga et al. [109] described a post-translational modification in the structure of Ts15 that presented an N-glycosylation; this was the first toxin in the *T. serrulatus* venom to have this modification described.

Ts16 toxin shows high selectivity towards blocking the Kv1.2 subtype of potassium channels, and this selectivity is demonstrated by means of two-electrode voltage-clamp technique [94]. This toxin demonstrated 62% of identity with Tt28, a component from the *T. trivittatus* venom, belonging to α-KTx20.1 [110].

Ts19 is a toxin that has been described initially from peptide fragments identified by peptidomic analyses. These fragments are related to β-KTx, toxins that act on potassium channels [94, 111]. Subsequently, transcriptome studies were able to identify the precursor sequence of this toxin, called Ts19 [95]. Currently, in the literature, there are three fragments related to Ts19, which are Ts19 Frag-I, Ts19 Frag-II and Ts19 Frag-III [64, 94]. The Ts19 fragment Frag-I shows 58 amino acid residues and has a high level of identity with toxins that act on potassium channels (KTx) [112]. In relation to the Ts19 Frag-II, it has 49 amino acid residues and was described as a β-KTx 2 toxin, characterized by an important selective and blocking action on Kv1.2 potassium channels [64, 113].

Finally, Ts14 represents a group of four peptides classified as hypotensins, TsHpt-I to TsHpt-IV [114]. The tests with TsHpt-I in rats in vivo demonstrated that this toxin has a bradykinin potentiating effect and a vasorelaxation effect on aortic rings dependent on nitric oxide [115].

### *Tityus bahiensis*

**Fig. 2:**
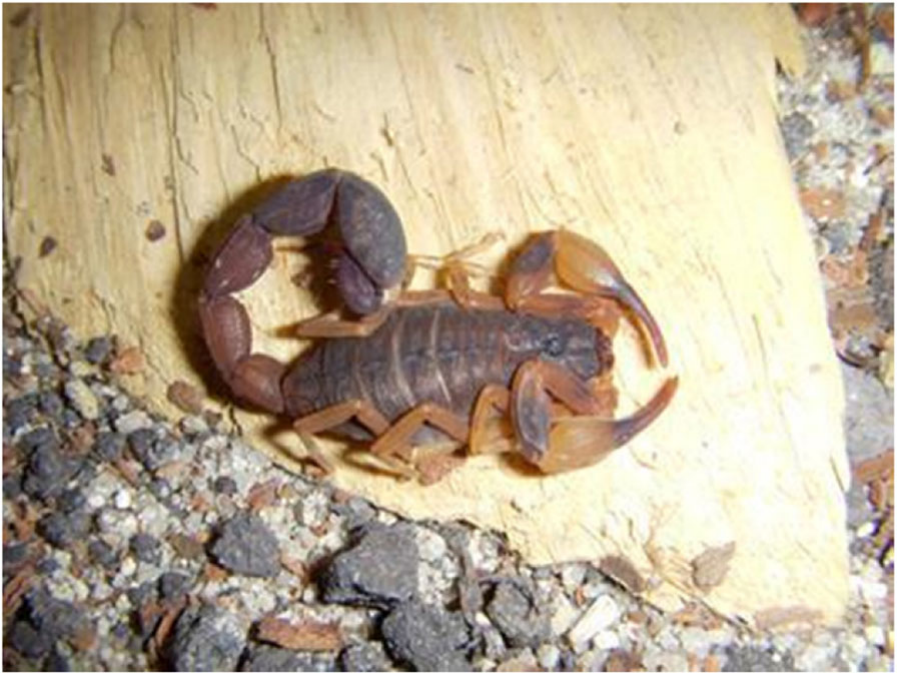
*Tityus bahiensis.* Known as the brown scorpion, *T. bahiensis* has a dark trunk, legs and palps with dark spots and reddish brown tail. The adult measures about 7 cm and presents sexual reproduction. Source: Brazilian Ministry of Health [6]. Image copyright by Denise Cândido, reproduced with permission

Unlike *T. serrulatus*, the distribution of *T. bahiensis* (Fig. 2) depends on its biological and ecological needs, such as sexual reproduction and relationship with environmental changes (including temperature and humidity), which limits its presence to the central and southwest regions of the state of Minas Gerais, the western São Paulo and northern Paraná [116].

*T. bahiensis* is responsible for most of the accidents in the Southeastern region of Brazil. However, in general, these accidents are considered mild with only local pain. In the literature, there is only one case of a patient with more severe symptoms and the involvement of the central nervous system [44].

Several experimental studies have been carried out with *T. bahiensis* venom, most of them dedicated to the purification and sequencing of the toxins [79, 117, 118]. Others aimed to describe the activity of some toxins, such as anti-insect or proteolytic properties, or the effects of the venom on sodium channels [119–121]. Recently, the transcriptome was performed in order to identify the main components of the venom [122].

Regarding the action on the central nervous system, it was demonstrated that crude venom, when peripherally injected into rats, promotes behavioral alterations such as wet dog shake, chewing movements, postural loss and sometimes priapism, as well as electrographic alterations including isolated spikes in the cortex and in the hippocampus [17]. Moreover, the intracerebral levels of homovanillic acid (HVA) are increased [17]. The partially purified venom, intravenously injected in mice, causes convulsion [18].

The direct application in the central nervous system of rats causes behavioral alterations such as wet dog shake, myoclonus and immobility and clonus of limbs, and electrographic alterations characterized by moderate and intense discharges, and neuronal loss in CA1, CA3 and CA4 hippocampal areas [18]. When the study is performed with purified toxins applied directly in the hippocampus of rats, the following alterations appear: wet dog shake, myoclonus, yawning, orofacial automatisms, and isolated or grouped spikes and epileptic-like discharges, varying in intensity from short to medium or strong [19, 123]. An increase in the extracellular level of glutamate and neuronal loss in the hippocampus are also observed as a consequence of the increase in the intracellular calcium concentration [19].

### *Tityus stigmurus*

**Fig. 3:**
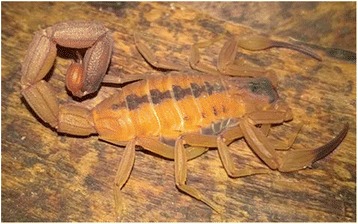
*Tityus stigmurus.* The yellow scorpion of the Northeast resembles *T. serrulatus* in habits and coloration, but it shows a dark longitudinal band in the dorsal area. Adult specimens are black and can reach 9 cm. They present sexual reproduction. Source: Brazilian Ministry of Health [6]. Image copyright by Denise Cândido, reproduced with permission

*T. stigmurus* (Fig. 3) is responsible for most of the accidents in the Brazilian Northeastern region [124]. Many of these accidents are mild, and the death of only three children with less than five years of age has been reported in the period from 2006 to 2010 in the state of Pernambuco [125]. The severity of the envenoming is similar to that caused by *T. serrulatus* and is characterized by pain, edema, erythema, paresthesia, headache and vomiting [126].

*T. stigmurus* venom has been further studied in the last few years by means of proteomic and transcriptomic approaches in order to characterize the genic expression of the venom gland [126, 127]. Several peptides of pharmacological interest have been identified including hypotensins, antimicrobial peptides and toxins active on sodium and potassium channels [126, 128, 129]. Other studies evaluated the effects of this venom on the renal function [130], analyzed the structure and toxicity of a hypotensive peptide [131], and characterized potassium channel blocker peptides [132, 133]. There are neither experimental studies nor clinical data demonstrating the central effect of this venom.

### *Tityus obscurus*

**Fig. 4:**
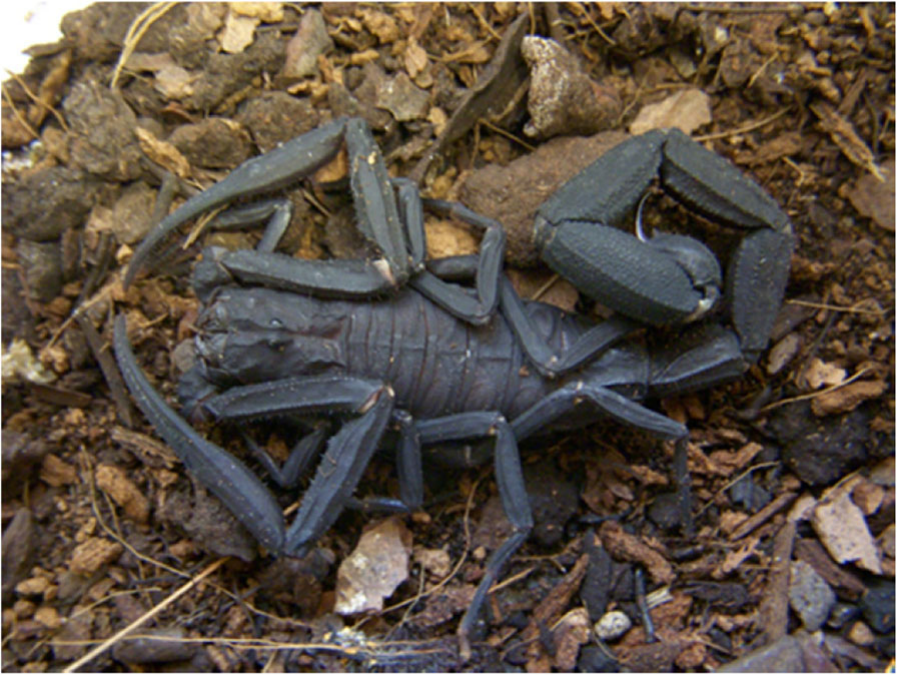
*Tityus obscurus*. Adults are black and can reach 9 cm in length. However, young animals are brown. They present sexual reproduction. Source: Brazilian Ministry of Health [6]. Image copyright by Denise Cândido, reproduced with permission

*T. obscurus* (Fig. 4), also known as *T. cambridgei* or *T. paraensis*, is the most dangerous found in the Amazon forest and it is responsible for several accidents in this region [134, 135].

The effects of their stings may be different according to the region of origin. Generally, a local and radiating pain is observed, as well as paresthesia, edema, erythema, sweating, piloerection and burning. Paresthesia and radiating pain predominated in patients from the western region of the state of Pará [136]. The main neurological effects are myoclonus, dysmetria and ataxia, without autonomic manifestations. Myoclonus, electric shock-like sensations in the body, dysarthria, paresthesia, ataxia and dysmetria were reported only in patients from the western region of the state of Pará [136]. It was reported that the vast majority of the patients presented symptoms compatible with acute cerebellar dysfunction and abnormal neuromuscular manifestations and, in some cases, muscle injury, which Torrez et al. [137] claim that have never been described in any other region of the world.

The composition of the venom is poorly known. The first studies characterizing its components started less the 20 years ago, when the complete description of a potassium channel blocker peptide was carried out and four new active toxins on the sodium channel were described [138, 139]. Other peptides specific for the potassium channel, particularly on the Kv1.3 channel, which is pivotal for the functioning of cells related to the immune system, were described later as well as sodium channel toxins [140–142]. Recently, the cDNA library of venom glands was built [134].

Experimentally, it was demonstrated that *T. obscurus* venom acts directly on skeletal muscle, differently from *T. serrulatus* venom [143]. It was also demonstrated that this venom causes hemorrhagic patches in the lung parenchyma, but it does not lead to pulmonary edema when intraperitoneally injected into rats, and promotes a decrease in the general activity without inducing convulsions neither hippocampal neuronal loss. In mice, it induces edematogenic and moderate nociceptive activity [144].

### Other *Tityus*

**Fig. 5:**
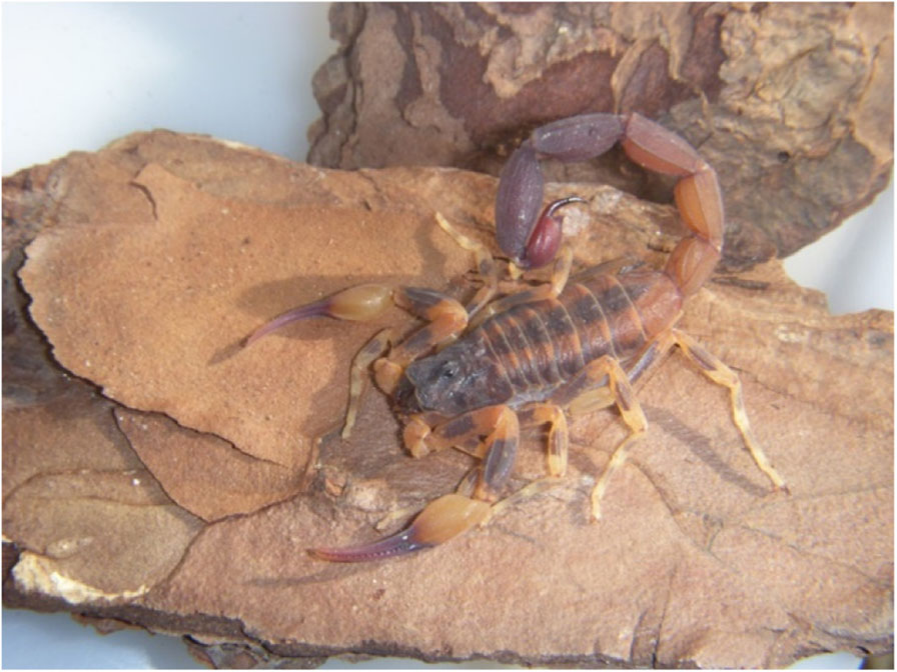
*Tityus fasciolatus.* It is generally yellowish brown with three longitudinal bands on the dorsal side of the trunk. It can measure from 4.5 to 7 cm in length. Source: Brazilian Ministry of Health [6]. Image copyright by Denise Cândido, reproduced with permission

*T. fasciolatus* scorpion (Fig. 5) is found mainly in the central region of Brazil, where it is responsible for some accidents [145]. Little information is available on the toxicity of this venom. The first pharmacological characterization of the venom was conducted in 2003, when a toxin active on the sodium channel was isolated [145]. The deleterious effect of the venom on the cardiovascular system was more recently determined, and other active toxins on the sodium channel were identified [146, 147]. Immunologically and molecularly, this venom was considered to be similar to *T. serrulatus* venom [148]. There is no information on its effect on the central nervous system.

**Fig. 6:**
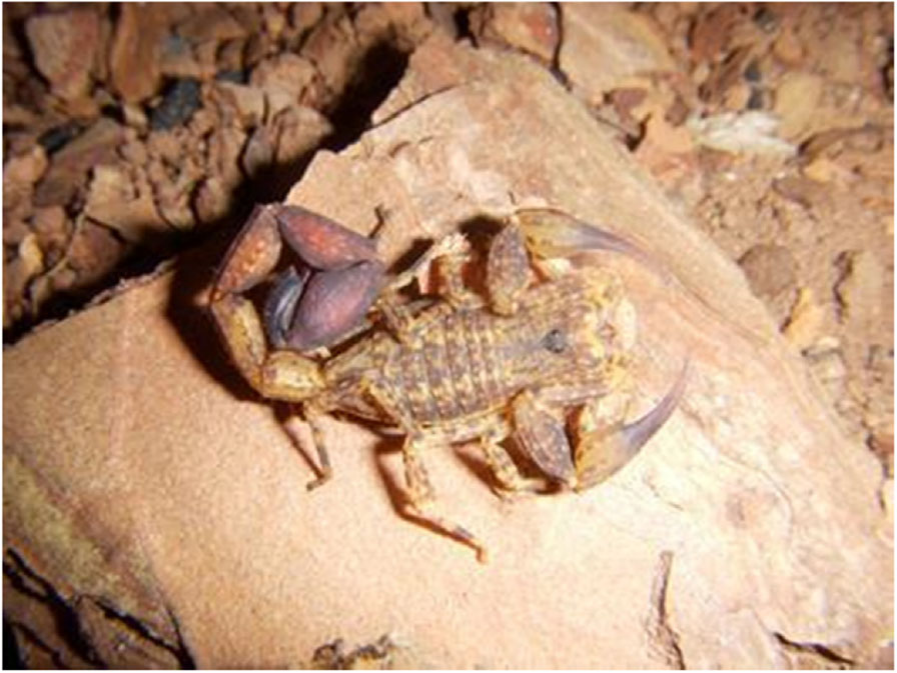
*Tityus silvestris.* It is generally yellowish brown with spots all over the body, legs and palps. It can measure from 2.5 to 4.5 cm in length. Source: Brazilian Ministry of Health [6]. Image copyright by Denise Cândido, reproduced with permission

Little information is available on another Amazonian scorpion, *T. silvestris* (Fig. 6). The first description of the systemic effects of its venom is recent [149]. Symptoms include nausea, vomiting, somnolence, malaise and prostration. Muscular spasms are described after the scorpion sting, classifying the case as severe envenoming [150].

**Fig. 7:**
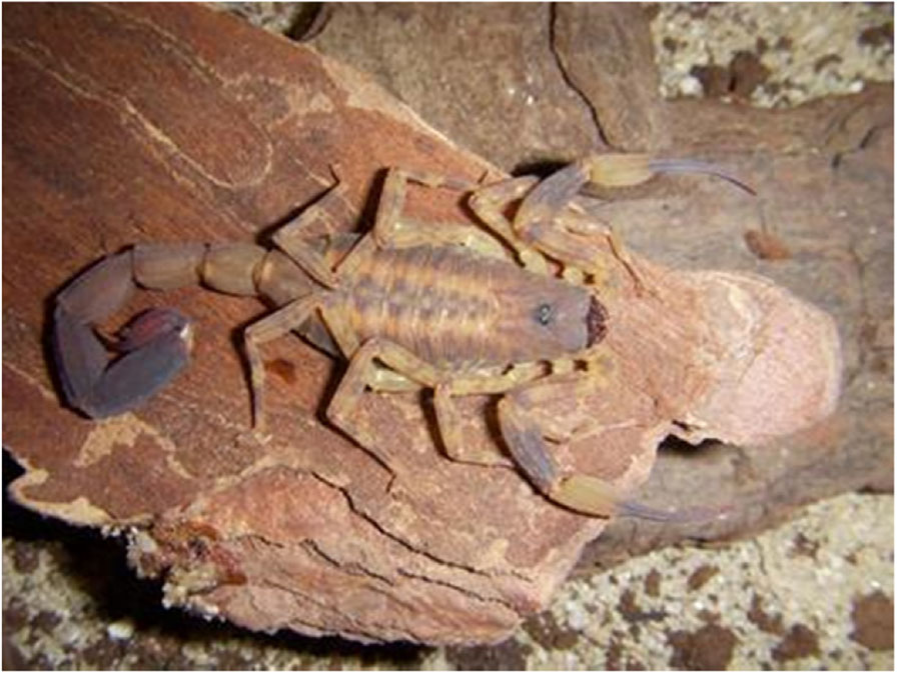
*Tityus costatus.* This species is yellowish brown with spots on the legs and palps. It can measure from 5 to 7 cm in length. Source: Brazilian Ministry of Health [6]. Image copyright by Denise Cândido, reproduced with permission

Regarding *T. costatus* (Fig. 7), there is only one study identifying some components of the venom, which are considered similar to those present in the scorpions of the *Tityus* genus [151].

## Conclusions

There are several evidences showing the direct participation of the central nervous system in the envenoming process provoked by scorpions. Although the central effects rarely appear in patients, they can be serious and potentially fatal, requiring special attention in the treatment of envenoming cases. In addition, these scorpion toxins may be important tools for central nervous system studies.

## References

[1] Prendini L, Wheeler WC (2005). Scorpion higher phylogeny and classification, taxonomic anarchy, and standards for peer review in online publishing. Cladistics.

[2] Chippaux JP, Goyffon M (2008). Epidemiology of scorpionism: a global appraisal. Acta Trop.

[3] Lourenço WR (2014). A historical approach to scorpion studies with special reference to the 20th and 21st centuries. J Venom Anim Toxins incl Trop Dis.

[4] Lourenço WR (2015). What do we know about some of the most conspicuous scorpion species of the genus *Tityus*? A historical approach. J Venom Anim Toxins incl Trop Dis.

[5] Lourenço WR (2016). Scorpion incidents, misidentification cases and possible implications for the final interpretation of results. J Venom Anim Toxins incl Trop Dis.

[6] Brasil. Ministério da Saúde. Manual de controle de escorpiões. 2009. p. 1–74. http://bvsms.saude.gov.br/bvs/publicacoes/manual_controle_escorpioes.pdf

[7] Pucca MB, Oliveira FN, Schwartz EF, Arantes EC, Lira-da-Silva RM. Scorpionism and dangerous species of Brazil. In: Gopalakrishnakone P, Possani LD, Schwartz EF, de la Vega RCR, editors. Scorpion Venoms. Springer Netherlands; 2015. p. 299–324. doi:10.1007/978-94-007-6404-0_20.

[8] Reckziegel GC, Pinto VL (2014). Scorpionism in Brazil in the years 2000 to 2012. J Venom Anim Toxins incl Trop Dis.

[9] Chippaux JP (2015). Epidemiology of envenomations by terrestrial venomous animals in Brazil based on case reporting: from obvious facts to contingencies. J Venom Anim Toxins incl Trop Dis.

[10] Bochner R. The international view of envenoming in Brazil: myths and realities. J Venom Anim Toxins incl Trop Dis. 2013;19:29. doi: 10.1186/1678-9199-19-29. 10.1186/1678-9199-19-29 PMC3842768 24215797

[11] Lourenço WR, Cloudsley-Thompson JL, Cuellar O, Von Eickstedt VRD, Barraviera B, Knox MB (1996). The evolution of scorpionism in Brazil in recent years. J Venom Anim Toxins incl Trop Dis.

[12] Brasil. Ministério da Saúde. Manual de diagnóstico e tratamento por animais peçonhentos. 2001. p. 1–111. http://bvsms.saude.gov.br/bvs/publicacoes/funasa/manu_peconhentos.pdf.

[13] Queiroz AM, Sampaio VS, Mendonça I, Fé NF, Sachett J, Ferreira LCL (2015). Severity of scorpion stings in the Western Brazilian Amazon: a case-control study. PLoS One.

[14] Dorce VA, Sandoval MR (1994). Effects of *Tityus serrulatus* crude venom on the GABAergic and dopaminergic systems of the rat brain. Toxicon.

[15] Carvalho FF, Nencioni ALA, Lebrun I, Sandoval MRL, Dorce VAC (1998). Behavioral, electroencephalographic and histopathologic effects of a neuropeptide isolated from *Tityus serrulatus* scorpion venom in rats. Pharmacol Biochem Behav.

[16] Nencioni ALA, Carvalho FF, Lebrun I, Dorce VAC, Sandoval MRL (2000). Neurotoxic effects of three fractions isolated from *Tityus serrulatus* scorpion venom. Pharmacol Toxicol.

[17] Nencioni ALA, Lourenço GA, Lebrun I, Florio JC, Dorce VAC (2009). Central effects of *Tityus serrulatus* and *Tityus bahiensis* scorpion venoms after intraperitoneal injection in rats. Neurosci Lett.

[18] Lourenço GA, Lebrun I, Dorce VAC (2002). Neurotoxic effects of fractions isolated from *Tityus bahiensis* scorpion venom (Perty, 1834). Toxicon.

[19] Ossanai LTT, Lourenço GA, Nencioni ALA, Lebrun I, Yamanouye N, Dorce VAC (2012). Effects of a toxin isolated from *Tityus bahiensis* scorpion venom on the hippocampus of rats. Life Sci.

[20] Pucca MB, Cerni FA, Pinheiro Junior EL, Bordon KCF, Amorim FG, Cordeiro FA (2015). *Tityus serrulatus* venom--A lethal cocktail. Toxicon.

[21] Barraviera B, Lomonte B, Tarkowski A, Hanson LA, Meira DA (1995). Acute-phase reactions, including cytokines, in patients bitten by *Bothrops* and *Crotalus* snakes in Brazil. J Venom Anim Toxins incl Trop Dis.

[22] Voronov E, Apte RN, Sofer S (1999). The systemic inflammatory response syndrome related to the release of cytokines following severe envenomation. J Venom Anim Toxins incl Trop Dis.

[23] Petricevich VL (2006). Balance between pro- and anti-inflammatory cytokines in mice treated with *Centruroides noxius* scorpion venom. Mediators Inflamm.

[24] Petricevich VL (2010). Scorpion venom and the inflammatory response. Mediators Inflamm.

[25] D'Suze G, Moncada S, González C, Sevcik C, Aguilar V, Alagón A (2003). Relationship between plasmatic levels of various cytokines, tumour necrosis factor, enzymes, glucose and venom concentration following *Tityus* scorpion sting. Toxicon.

[26] Fukuhara YD, Reis ML, Dellalibera-Joviliano R, Cunha FQ, Donadi EA (2003). Increased plasma levels of IL-1β, IL-6, IL-8, IL-10 and TNF-α in patients moderately or severely envenomed by *Tityus serrulatus* scorpion sting. Toxicon.

[27] Zoccal KF, Bitencourt CS, Secatto A, Sorgi CA, Bordon KD, Sampaio SV (2011). *Tityus serrulatus* venom and toxins Ts1, Ts2 and Ts6 induce macrophage activation and production of immune mediators. Toxicon.

[28] Zoccal KF, Bitencourt CS, Sorgi CA, Bordon KD, Sampaio SV, Arantes EC (2013). Ts6 and Ts2 from *Tityus serrulatus* venom induce inflammation by mechanisms dependent on lipid mediators and cytokine production. Toxicon.

[29] Zoccal KF, Paula-Silva FW, Bitencourt CS, Sorgi CA, Bordon KD, Arantes EC (2015). PPAR-γ activation by *Tityus serrulatus* venom regulates lipid body formation and lipid mediator production. Toxicon.

[30] Zoccal KF, Sorgi CA, Hori JI, Paula-Silva FW, Arantes EC, Serezani CH (2016). Opposing roles of LTB4 and PGE2 in regulating the inflammasome-dependent scorpion venom-induced mortality. Nat Commun.

[31] Freire-Maia L, Campos JA. Pathophysiology and treatment of scorpion poisoning. In: Ownby CL, Odell GV, editors. Natural Toxins, Characterization, Pharmacology and Therapeutics. Oxford: Pergamon Press; 1989. ISBN 0–08–036139-0.

[32] Ismail M, Abd-Elsalam MA, Morad AM (1990). Do changes in body temperature following envenomation by the scorpion *Leiurus quinquestriatus* influence the course of toxicity?. Toxicon.

[33] Ismail M, Fatani AJY, Dabees TT (1992). Experimental treatment protocols for scorpion envenomation: a review of common therapies and an effect of kallikrein-kinin inhibitors. Toxicon.

[34] Nagaraja D, Verma A, Taly AB, Kumar MV, Jayakumar PN (1994). Cerebrovascular disease in children. Acta Neurol Scand.

[35] Barthwal SP, Agarwal R, Kanna D, Dwivedi NC, Agarwal DK (1997). Myocarditis and hemiplegia from scorpion bite--a case report. Indian J Med Sci.

[36] Fernández-Bouzas A, Morales-Reséndiz ML, Llamas-Ibarra F, Martínez-López M, Ballesteros-Maresma A (2000). Brain infarcts due to scorpion stings in children: MRI. Neuroradiol.

[37] Tracker AK, Lal R, Misra M (2002). Scorpion bite and multiple cerebral infarcts. Neurol India.

[38] Gadwlakar SR, Bushan S, Pramod K, Gouda C, Kumar PM (2006). Bilateral cerebellar infarction: a rare complication of scorpion sting. J Assoc Physicians India.

[39] Prasad R, Suri S, Shambhavi Mishra OMP (2014). Ischemic infarction of cerebral cortex in a child with scorpion sting envenomation. Indian J Pediatr.

[40] Sigirci A, Öztürk M, Yakinci C. Cerebral atrophy and subdural haemorrhage after cerebellar and cerebral infarcts in an 8-month-old child after having been stung by a scorpion. BMJ Case Rep. 2014;2014:bcr2014205091. doi:10.1136/bcr-2014-205091. 10.1136/bcr-2014-205091 PMC4069766 24962491

[41] Bonilha L, Cendes F, Ghizoni E, Vieira RJ, Li LM (2004). Epilepsy due to a destructive brain lesion caused by a scorpion sting. Arch Neurol..

[42] Oliveira DFB, Campos GS, Silveira LR, Faleiro RM, Yuami LN, Campolina D (2009). Acidente vascular encefálico unilateral consequente a acidente escorpiônico grave: relato de caso. Rev Bras Toxicol.

[43] Souza DG, Tanaka K, Algemiro W, Dezena RA, Borges MM, Pereira CU (2013). Hemorrhagic stroke following scorpion sting. A case report. Rev Chil Neurocirugía.

[44] Marrone LCP, Marrone BF, Kalil Neto F, Costa FC, Thomé GG, Aramburu MB (2013). Posterior reversible encephalopathy syndrome following a scorpion sting. J Neuroimaging.

[45] Bucaretchi F, De Capitani EM, Fernandes CB, Santos TM, Zamilute IAG, Hyslop S (2016). Fatal ischemic stroke following *Tityus serrulatus* scorpion sting in a patient with essential thrombocythemia. Clin Toxicol (Phila).

[46] Teixeira VF, Conceição IM, Lebrun I, Nencioni AL, Coronado Dorce VA (2010). Intrahippocampal injection of TsTX-I, a beta-scorpion toxin, causes alterations in electroencephalographic recording and behavior in rats. Life Sci.

[47] Bucaretchi F, Fernandes LCR, Fernandes CB, Branco MM, Prado CC, Vieira RJ (2014). Clinical consequences of *Tityus bahiensis* and *Tityus serrulatus* scorpion stings in the region of Campinas, southeastern Brazil. Toxicon.

[48] Santos MSV, Silva CGL, Silva Neto B, Grangeiro Junior CRP, Lopes VHG, Teixeira Junior AG (2016). Clinical and epidemiological aspects of scorpionism in the world: a systematic review. Wilderness Environ Med.

[49] Gomez MV, Diniz CR (1966). Separation of toxic components from the Brazilian scorpion - *Tityus serrulatus* - venom. Mem Inst Butantan.

[50] Arantes EC, Sampaio SV, Vieira CA, Giglio JR (1992). What is tityustoxin?. Toxicon.

[51] Sampaio SV, Laure CJ, Giglio JR (1983). Isolation and characterization of toxic proteins from the venom of the Brazilian scorpion *Tityus serrulatus*. Toxicon.

[52] Arantes EC, Prado WA, Sampaio SV, Giglio JR (1989). A simplified procedure for the fractionation of *Tityus serrulatus* venom: isolation and partial characterization of TsTX-IV, a new neurotoxin. Toxicon.

[53] Sampaio SV, Arantes EC, Prado WA, Riccioppo Neto F, Giglio JR (1991). Further characterization of toxins T1IV (TsTX-III) and T2IV from *Tityus serrulatus* scorpion venom. Toxicon.

[54] Lima EG, Almeida HO, Gomez MV, Freire-Maia L (1975). Acute pulmonary edema induced by injection of tityustoxin into the lateral ventricles of rats. Toxicon.

[55] Marangoni S, Ghiso J, Sampaio SV, Arantes EC, Giglio JR, Oliveira B (1990). The complete amino acid sequence of toxin TsTX-VI isolated from the venom of the scorpion *Tityus serrulatus*. J Protein Chem.

[56] Mansuelle P, Martin-Eauclaire MF, Chavez-Olortegui C, de Lima ME, Rochat H, Granier C (1992). The β-Type toxin Ts II from the scorpion *Tityus serrulatus*: amino acid sequence determination and assessment of biological and antigenic properties. Nat Toxins.

[57] Arantes EC, Riccioppo-Neto F, Sampaio SV, Vieira CA, Giglio JR (1994). Isolation and characterization of TsTX-V, a new neurotoxin from *Tityus serrulatus* scorpion venom which delays the inactivation of Na^+^ channels. Biochim Biophys Acta.

[58] Marangoni S, Toyama MH, Arantes EC, Giglio JR, da Silva CA, Carneiro EM (1995). Amino acid sequence of TsTX-V, an alpha-toxin from *Tityus serrulatus* scorpion venom, and its effect on K+ permeability of beta-cells from isolated rat islets of Langerhans. Biochim Biophys Acta.

[59] Blanc E, Lecomte C, Van Rietschoten J, Sabatier JM, Darbon H (1997). Solution structure of Tskapa, a charybdotoxin-like scorpion toxin from *Tityus serrulatus* with high affinity for apamin-sensitive Ca(2+)-activated K+ channels. Proteins.

[60] Cologna CT, Peigneur S, Rosa JC, Selistre-de-Araujo HS, Varanda WA, Tytgat J (2011). Purification and characterization of Ts15, the first member of a new α-KTX subfamily from the venom of the Brazilian scorpion *Tityus serrulatus*. Toxicon.

[61] Coelho VA, Cremonez CM, Anjolette FA, Aguiar JF, Varanda WA, Arantes EC (2014). Functional and structural study comparing the C-terminal amidated β-neurotoxin Ts1 with its isoform Ts1-G isolated from *Tityus serrulatus* venom. Toxicon.

[62] Pucca MB, Peigneur S, Cologna CT, Cerni FA, Zoccal KF, Bordon KCF (2015). Electrophysiological characterization of the first *Tityus serrulatus* alpha-like toxin, Ts5: Evidence of a pro-inflammatory toxin on macrophages. Biochimie.

[63] Pucca MB, Cerni FA, Peigneur S, Bordon KCF, Tytgat J, Arantes EC (2015). Revealing the function and the structural model of Ts4: insights into the “non-toxic” toxin from *Tityus serrulatus* venom. Toxins (Basel).

[64] Cerni FA, Pucca MB, Amorim FG, Bordon KCF, Echterbille J, Quinton L (2016). Isolation and characterization of Ts19 Fragment II, a new long-chain potassium channel toxin from *Tityus serrulatus* venom. Peptides.

[65] Revelo MP, Bambirra EA, Ferreira AP, Diniz CR, Chávez-Olórtegui C (1996). Body distribution of *Tityus serrulatus* scorpion venom in mice and effects of scorpion antivenom. Toxicon.

[66] Nunan EA, Moraes MFD, Cardoso VN, Moraes-Santos T (2003). Effect of age on body distribution of Tityustoxin from *Tityus serrulatus* scorpion venom in rats. Life Sci.

[67] Nunan EA, Arya V, Hochhaus G, Cardoso VN, Moraes-Santos T (2004). Age effects on the pharmacokinetics of tityustoxin from *Tityus serrulatus* scorpion venom in rats. Braz J Med Biol Res.

[68] Guidine PAM, Mesquita MBS, Moraes-Santos T, Massensini AR, Moraes MFD (2009). Electroencephalographic evidence of brainstem recruitment during scorpion envenomation. Neurotoxicology.

[69] Guidine PAM, Cash D, Drumond LE (2014). de Souza e Rezende GH, Massensini AR, Williams SCR, et al. Brainstem structures are primarily affected in an experimental model of severe scorpion envenomation. Toxicol Sci.

[70] Rhoads DE, Peterson NA, Sankaran H, Raghupathy E (1982). Inhibitory effects of scorpion venom on the uptake of amino acids by synaptosomes and synaptosomal membrane vesicles. Biochem Pharmacol.

[71] Sandoval MRL, Dorce VAC (1993). Behavioural and electroencephalographic effects of *Tityus serrulatus* scorpion venom in rats. Toxicon.

[72] Cecchini AL, Vasconcelos F, Amara SG, Giglio JR, Arantes EC (2006). Effects of *Tityus serrulatus* scorpion venom and its toxin TsTX-V on neurotransmitter uptake *in vitro*. Toxicol Appl Pharmacol.

[73] Corrêa MM, Sampaio SV, Lopes RA, Mancuso LC, Cunha OAB, Franco JJ (1997). Biochemical and histopathological alterations induced in rats by *Tityus serrulatus* scorpion venom and its major neurotoxin Tityustoxin-I. Toxicon.

[74] Vasconcelos F, Lanchote VL, Bendhack LM, Giglio JR, Sampaio SV, Arantes EC (2005). Effects of voltage-gated Na^+^ channel toxins from *Tityus serrulatus* venom on rat arterial blood pressure and plasma catecholamines. Comp Biochem Physiol C Toxicol Pharmacol.

[75] Cologna CT, Marcussi S, Giglio JR, Soares AM, Arantes EC (2009). *Tityus serrulatus* scorpion venom and toxins: an overview. Protein Pept Lett.

[76] Peigneur S, Cologna CT, Cremonez CM, Mille BG, Pucca MB, Cuypers E (2015). A gamut of undiscovered electrophysiological effects produced by *Tityus serrulatus* toxin 1 on NaV-type isoforms. Neuropharmacology.

[77] Rodriguez RV, Dorce VAC, de Freitas LA, Dorce ALC, Lebrun I, Sobral ACM (2015). Intrahippocampal injection of TsTX-I increases the levels of INF-γ in the cerebral tissue but not the levels of glutamate. Toxicon.

[78] Van Fraga IT, Limborço-Filho M, Lima OCO, Lacerda-Queiroz N, Guidine PAM, Moraes MFD (2015). Effects of tityustoxin on cerebral inflammatory response in young rats. Neurosci Lett.

[79] Becerril B, Marangoni S, Possani LD (1997). Toxins and genes isolated from scorpions of the genus *Tityus*. Toxicon.

[80] Kalapothakis E, Chávez-Olórtegui C (1997). Venom variability among several *Tityus serrulatus* specimens. Toxicon.

[81] Guidine PA, Moraes-Santos T, Massensini AR, Moraes MF (2008). Carbamazepine protects the CNS of Wistar rats against the central effects of scorpion envenomation. Neurotoxicology.

[82] Mesquita MB, Moraes-Santos T, Moraes MF (2003). Centrally injected tityustoxin produces the systemic manifestations observed in severe scorpion poisoning. Toxicol Appl Pharmacol.

[83] Barhanin J, Giglio JR, Léopold P, Schmid A, Sampaio SV, Lazdunski M (1982). *Tityus serrulatus* venom contains two classes of toxins. *Tityus* gamma toxin is a new tool with a very high affinity for studying the Na^+^ channel. J Biol Chem.

[84] Casali TA, Gomez RS, Moraes-Santos T, Gomez MV (1995). Differential effects of calcium channel antagonists on tityustoxin and ouabain-induced release of [^3^H] acetylcholine from brain cortical slices. Neuropharmacology.

[85] Massensini AR, Moraes-Santos T, Gomez MV, Romano-Silva MA (1998). Alpha- and beta-scorpion toxins evoke glutamate release from rat cortical synaptosomes with different effects on [Na^+^]i and [Ca^2+^]i. Neuropharmacology.

[86] Nencioni ALA, Lebrun I, Dorce VAC (2003). A microdialysis study of glutamate concentration in the hippocampus of rats after TsTX toxin injection and blockade of toxin effects by glutamate receptor antagonists. Pharmacol Biochem Behav.

[87] Nencioni ALA, Lebrun I, Dorce VAC (2004). Dantrolene protects hippocampal cells from damage induced by TsTX, an α-scorpion toxin from *Tityus serrulatus*. Toxicon.

[88] Sandoval MR, Lebrun I (2003). TSII toxin isolated from *Tityus serrulatus* scorpion venom: behavioral, electroencephalographic, and histopathologic studies. Brain Res Bull.

[89] Fletcher PL, Fletcher M, Fainter LK, Terrian DM (1996). Action of a new word scorpion venom and its neurotoxins in secretion. Toxicon.

[90] Silva FC, Guidine PA, Machado NL, Xavier CH, de Menezes RC, Moraes-Santos T (2015). The role of dorsomedial hypotalamus ionotropic glutamate receptors in the hypertensive and tachycardic responses evoked by Tityustoxin intracerebroventricular injection. Neurotoxicology.

[91] Mesquita MB, Moraes-Santos T, Moraes MF (2002). Phenobarbital blocks the lung edema induced by centrally injected tityustoxin in adult Wistar rats. Neurosci Lett.

[92] Sampaio SV, Coutinho-Netto J, Arantes EC, Marangoni S, Oliveira B, Giglio JR (1996). Isolation of toxin TsTX-VI from *Tityus serrulatus* scorpion venom. Effects on the release of neurotransmitters from synaptosomes. Biochem Mol Biol Int.

[93] Cologna CT, Peigneur S, Rustiguel JK, Nonato MC, Tytgat J, Arantes EC (2012). Investigation of the relationship between the structure and function of Ts2, a neurotoxin from *Tityus serrulatus* venom. FEBS J.

[94] Bordon KCF, Cologna CT, Arantes EC. Scorpion venom research around the World: *Tityus serrulatus*. In: Gopalakrishnakone P, Possani LD, Schwartz EF, de la Vega RCR, editors. Scorpion Venoms. Springer Netherlands; 2015. p. 411–438. doi: 10.1007/978-94-007-6404-0_7.

[95] Alvarenga ER, Mendes TM, Magalhaes BF, Siqueira FF, Dantas AE, Barroca TM (2012). Transcriptome analysis of the *Tityus serrulatus* scorpion venom gland. Open J Genet.

[96] Morgenstern D, Rohde BH, King GF, Tal T, Sher D, Zlotkin E (2011). The tale of a resting gland: transcriptome of a replete venom gland from the scorpion *Hottentotta judaicus*. Toxicon.

[97] Novello JC, Arantes EC, Varanda WA, Oliveira B, Giglio JR, Marangoni S (1999). TsTX-IV, a short chain four-disulfide-bridged neurotoxin from *Tityus serrulatus* venom which acts on Ca^2+^-activated K^+^ channels. Toxicon.

[98] Cerni FA, Pucca MB, Peigneur S, Cremonez CM, Bordon KCF, Tytgat J (2014). Electrophysiological characterization of Ts6 and Ts7, K+ channel toxins isolated through an improved *Tityus serrulatus* venom purification procedure. Toxins (Basel).

[99] Oyama S, Pristovsek P, Franzoni L, Pertinhez TA, Schinina E, Lucke C (2005). Probing the pH-dependent structural features of alpha-KTx12.1, a potassium channel blocker from the scorpion Tityus serrulatus. Protein Sci.

[100] Eccles CU, Rogowski RS, Gu X, Alger BE, Blaustein MP (1994). Tityustoxin-K alpha, from scorpion venom, blocks voltage-gated, non-inactivating potassium current in cultured central neurons. Neuropharmacology.

[101] Rogowski RS, Krueger BK, Collins JH, Blaustein MP (1994). Tityustoxin K alpha blocks voltage-gated noninactivating K^+^ channels and unblocks inactivating K+ channels blocked by alpha-dendrotoxin in synaptosomes. Proc Natl Acad Sci U S A.

[102] Matteson DR, Blaustein MP (1997). Scorpion toxin block of the early K+ current (*I*Kf) in rat dorsal root ganglion neurones. J Physiol.

[103] Rodrigues AR, Arantes EC, Monje F, Stühmer W, Varanda WA (2003). Tityustoxin-K(alpha) blockade of the voltage-gated potassium channel Kv1.3. Br J Pharmacol.

[104] Werkman TR, Gustafson TA, Rogowski RS, Blaustein MP, Rogawski MA (1993). Tityustoxin-K alpha, a structurally novel and highly potent K^+^ channel peptide toxin, interacts with the alpha-dendrotoxin binding site on the cloned Kv1.2 K^+^ channel. Mol Pharmacol.

[105] Pucca MB, Cerni FA, Cordeiro FA, Peigneur S, Cunha TM, Tytgat J (2016). Ts8 scorpion toxin inhibits the Kv4. 2 channel and produces nociception *in vivo*. Toxicon.

[106] Pimenta AM, Legros C, Almeida Fde M, Mansuelle P, de Lima ME, Bougis PE (2003). Novel structural class of four disulfide-bridged peptides from *Tityus serrulatus* venom. Biochem Biophys Res Commun.

[107] Cremonez CM, Maiti M, Peigneur S, Cassoli JS, Dutra AA, Waelkens E, et al. Structural and functional elucidation of peptide Ts11 shows evidence of a novel subfamily of scorpion venom toxins. Toxins (Basel). 2016;8(10):pii: E288. 10.3390/toxins8100288 PMC5086648 27706049

[108] Pucca MB, Bertolini TB, Cerni FA, Bordon KC, Peigneur S, Tytgat J (2016). Immunosuppressive evidence of *Tityus serrulatus* toxins Ts6 and Ts15: insights of a novel K(+) channel pattern in T cells. Immunology.

[109] Verano-Braga T, Dutra AAA, León IR, Melo-Braga MN, Roepstorff P, Pimenta AMC (2013). Moving pieces in a venomic puzzle: unveiling post-translationally modified toxins from *Tityus serrulatus*. J Proteome Res.

[110] Abdel-Mottaleb Y, Coronas FV, de Roodt AR, Possani LD, Tytgat J (2006). A novel toxin from the venom of the scorpion *Tityus trivittatus* is the first member of a new alpha-KTX subfamily. FEBS Lett.

[111] Rates B, Ferraz KK, Borges MH, Richardson M, de Lima ME, Pimenta AM (2008). *Tityus serrulatus* venom peptidomics: assessing venom peptide diversity. Toxicon.

[112] Lima PC, Bordon KCF, Pucca MB, Cerni FA, Zoccal KF, Faccioli LH (2015). Partial purification and functional characterization of Ts19 Frag-I, a novel toxin from *Tityus serrulatus* scorpion venom. J Venom Anim Toxins incl Trop Dis.

[113] Cerni FA, Pucca MB, Zoccal KF, Frantz FG, Faccioli LH, Arantes EC (2017). Expanding biological activities of Ts19 Frag-II toxin: insights into IL-17 production. Toxicon.

[114] Verano-Braga T, Rocha-Resende C, Silva DM, Ianzer D, Martin-Eauclaire MF, Bougis PE (2008). *Tityus serrulatus* Hypotensins: a new family of peptides from scorpion venom. Biochem Biophys Res Commun.

[115] Verano-Braga T, Figueiredo-Rezende F, Melo MN, Lautner RQ, Gomes ER, Mata-Machado LT (2010). Structure-function studies of *Tityus serrulatus* Hypotensin-I (TsHpt-I): a new agonist of B(2) kinin receptor. Toxicon.

[116] Brites-Neto J, Duarte KMR (2015). Modeling of spatial distribution for scorpions of medical importance in the São Paulo State. Brazil. Vet World.

[117] Becerril B, Corona M, Corona FIV, Zamudio F, Calderon-Aranda FS, Fletcher PL (1996). Toxic peptides and genes encoding toxin gamma of the Brazilian scorpions *Tityus bahiensis* and *Tityus stigmurus*. Biochem J.

[118] Trequattrini C, Zamudio FZ, Petris A, Prestipino G, Possani LD, Franciolini F (1995). *Tityus bahiensis* toxin IV-5b selectively affects Na channel inactivation in chick dorsal root ganglion neurons. Comp Biochem Physiol A Physiol.

[119] Pimenta AMC, Martin-Eauclaire MF, Rochat H, Figueiredo SG, Kalapothakis E, Afonso LCC (2001). Purification, amino-acid sequence and partial characterization of two toxins with anti-insect activity from the venom of the South American scorpion *Tityus bahiensis* (Buthidae). Toxicon.

[120] Almeida FM, Pimenta ACM, de Figueiredo SG, Santoro MM, Martin-Eauclaire MF, Diniz CR (2002). Enzymes with gelatinolytic activity can be found in *Tityus bahiensis* and *Tityus serrulatus* venoms. Toxicon.

[121] Moraes ER, Kalapothakis E, Naves LA, Kushmerick C (2011). Differential effects of *Tityus bahiensis* scorpion venom on tetrodotoxin-sensitive and tetrodotoxin-resistant sodium currents. Neurotox Res.

[122] de Oliveira UC, Candido DM, Dorce VAC, Junqueira-de-Azevedo ILM (2015). The transcriptome recipe for the venom cocktail of *Tityus bahiensis* scorpion. Toxicon.

[123] Ossanai LTT, Lourenço GA, Lebrun I, Nencioni ALA, Dorce VAC. Convulsive and neurodegenerative effects in rats of some isolated toxins from the *Tityus bahiensis* scorpion venom. J Toxins. 2013;(2013):Article ID 501876. doi: 10.2013/501876.

[124] Albuquerque CMR, Barbosa MO, Iannuzzi L (2009). *Tityus stigmurus* (Thorell, 1876) (Scorpiones; Buthidae): response to chemical control and understanding of scorpionism among the population. Rev Soc Bras Med Trop.

[125] de Albuquerque CMR, Santana Neto PL, Amorim MLP, Pires SCV (2013). Pediatric epidemiological aspects of scorpionism and report on fatal cases from *Tityus stigmurus* stings (Scorpiones: Buthidae) in State of Pernambuco. Brazil. Rev Soc Bras Med Trop.

[126] Almeida DD, Scortecci KC, Kobashi LS, Agnez-Lima LF, Medeiros SRB, Silva-Júnior AA (2012). Profiling the resting venom gland of the scorpion *Tityus stigmurus* through a transcriptomic survey. BMC Genomics.

[127] Batista CVF, Román-González SA, Salas-Castillo SP, Zamudio FZ, Gómez-Lagunas F, Possani LD. Proteomic analysis of the venom from the scorpion *Tityus stigmurus*: biochemical and physiological comparison with other *Tityus* species. Comp Biochem Physiol C Toxicol Pharmacol. 2007;146(1–2):147–57. 10.1016/j.cbpc.2006.12.004 17270501

[128] Machado RJA, Junior LGM, Monteiro NKV, Silva-Júnior AA, Portaro FCV, Barbosa EG (2015). Homology modeling, vasorelaxant and bradykinin-potentiating activities of a novel hypotensin found in the scorpion venom from *Tityus stigmurus*. Toxicon.

[129] de Melo ET, Estrela AB, Santos ECG, Machado PRL, Farias KJS, Torres TM (2015). Structural characterization of a novel peptide with antimicrobial activity from the venom gland of the scorpion *Tityus stigmurus:* Stigmurin. Peptides.

[130] Silva NA, Albuquerque CMR, Marinho AD, Jorge RJB, Silva AG (2016). Neto, Monteiro HSA, et al. Effects of *Tityus stigmurus* (Thorell 1876) (Scorpiones: Buthidae) venom in isolated perfused rat kidneys. An Acad Bras Cienc.

[131] Machado RJA, Estrela AB, Nascimento AKL, Melo MMA, Torres-Rêgo M, Lima EO (2016). Characterization of TistH, a multifunctional peptide from the scorpion *Tityus stigmurus*: structure, cytotoxicity and antimicrobial activity. Toxicon.

[132] Papp F, Batista CVF, Varga Z, Herceg M, Román-González SA, Gaspar R (2009). Tst26, a novel peptide blocker of Kv1.2 and Kv1.3 channels from the venom of *Tityus stigmurus*. Toxicon.

[133] Almeida DD, Torres TM, Barbosa EG, Lima JPMS, Fernandes-Pedrosa MF (2013). Molecular approaches for structural characterization of a new potassium channel blocker from *Tityus stigmurus* venom: cDNA cloning, homology modeling, dynamic simulations and docking. Biochem Biophys Res Commun.

[134] Guerrero-Vargas JA, Mourão CBF, Quintero-Hernández V, Possani LD, Schwartz EF (2012). Identification and phylogenetic analysis of *Tityus pachyurus* and *Tityus obscurus* novel putative Na^+^-channel scorpion toxins. PLoS One.

[135] Asano ME, Arnund RM, Lopes FOB, Pardal JSO, Pardal PPO. Estudo clínico e epidemiológico de 12 acidentes por escorpiões atendidos no Hospital Universitário João de Barros Barreto, Belém-Pará, no período de 1992–1995. Rev Soc Bras Med Trop. 1996;29(Supl. I):243.

[136] Pardal PPO, Ishikawa EAY, Vieira JLF, Coelho JS, Dórea RCC, Abati PAM (2014). Clinical aspects of envenomation caused by *Tityus obscurus* (Gervais, 1843) in two distinct regions of Pará state. J Venom Anim Toxins incl Trop Dis.

[137] Torrez PPQ, Quiroga MMM, Abati PAM, Mascheretti M, Costa WS, Campos LP (2015). Acute cerebellar dysfunction with neuromuscular manifestations after scorpionism presumably caused by *Tityus obscurus* in Santarem. Para/Brazil. Toxicon.

[138] Batista CVF, Gómez-Lagunas F, Lucas S, Possani LD (2000). Tc1, from *Tityus cambridgei*, is the first member of a new subfamily of scorpion toxin that blocks K+−channels. FEBS Lett.

[139] Batista CVF, Zamudio FZ, Lucas S, Fox JW, Frau A, Prestipino G (2002). Scorpion toxins from *Tityus cambridgei* that affect Na^+^-channels. Toxicon.

[140] Batista CVF, Gómez-Lagunas F (2002). Rodríguez de la Vega RC, Hajdu P, Panyi G, Gáspár R, et al. Two novel toxins from the Amazonian scorpion *Tityus cambridgei* that block Kv1.3 and Shaker B K^+^-channels with distinctly different affinities. Biochim Biophys Acta.

[141] Batista CVF, del Pozo L, Zamudio FZ, Contreras S, Becerril B, Wanke E (2004). Proteomics of the venom from the Amazonian scorpion *Tityus cambridgei* and the role of prolines on mass spectrometry analysis of toxins. J Chromatogr B Analyt Biomed Life Sci.

[142] Murgia AR, Batista CVF, Prestipino G, Possani LD (2004). Amino acid sequence and function of a new a-toxin from the Amazonian scorpion *Tityus cambridgei*. Toxicon.

[143] Borja-Oliveira CR, Pertinhez TA, Rodrigues-Simioni L, Spisni A (2009). Positive inotropic effects of *Tityus cambridgei* and *T. serrulatus* scorpion venoms on skeletal muscle. Comp Biochem Physiol C Toxicol Pharmacol.

[144] Santos-da-Silva AP, Candido DM, Nencioni ALA, Kimura LF, Prezotto-Neto JP, Barbaro KC (2017). Some pharmacological effects of *Tityus obscurus* venom in rats and mice. Toxicon.

[145] Wagner S, Castro MS, Barbos JARG, Fontes W, Schwartz ENF, Sebben A (2003). Purification and primary structure determination of Tf4, the first bioactive peptide isolated from the venom of the Brazilian scorpion *Tityus fasciolatus*. Toxicon.

[146] Pinto MCL, Borboleta LR, Melo MB, Labarrére CR, Melo MM (2010). *Tityus fasciolatus* envenomation induced cardio-respiratory alterations in rats. Toxicon.

[147] Camargos TS, Bosmans F, Rego SC, Mourão CBF, Schwartz EF. The scorpion toxin Tf2 from *Tityus fasciolatus* promotes Nav1.3 opening. PLoS One. 2015;10(6):e0128578. doi: 10.1371/journal.pone.0128578. 10.1371/journal.pone.0128578 PMC4470819 26083731

[148] Mendes TM, Guimarães-Okamoto PTC, Machado-de-Avila RA, Oliveira D, Melo MM, Lobato ZI (2015). General characterization of *Tityus fasciolatus* scorpion venom. Molecular identification of toxins and localization of linear B-cell epitopes. Toxicon.

[149] Coelho JS, Ishikawa EAY, dos Santos PRSG, Pardal PPO (2016). Scorpionism by *Tityus silvestris* in eastern Brazilian Amazon. J Venom Anim Toxins incl Trop Dis.

[150] Monteiro WM, de Oliveira SS, Pivoto G, Alves EC, Sachett JAG, Alexandre CN (2016). Scorpion envenoming caused by *Tityus cf. silvestris* evolving with severe muscle spasms in the Brazilian Amazon. Toxicon.

[151] Diego-García E, Batista CVF, García-Gómez BI, Lucas S, Candido DM, Gómez-Lagunas F (2005). The Brazilian scorpion *Tityus costatus* Karsch: genes, peptides and function. Toxicon..

